# Supplementary material for: Splice-Junction-Based Mapping of Alternative Isoforms in the Human Proteome
Source: Cell Rep. Author manuscript; Available in PMC 2020 Jan 15. (PMC6961840; doi:10.1016/j.celrep.2019.11.026)
Supplement: 3 [file NIHMS1546469-supplement-3.zip › DF2/PXD0010154/Testis-169-Q14498-LAEGTGLQIPPAAQQALQMSGSLAFGAVADLQTR.pdf]

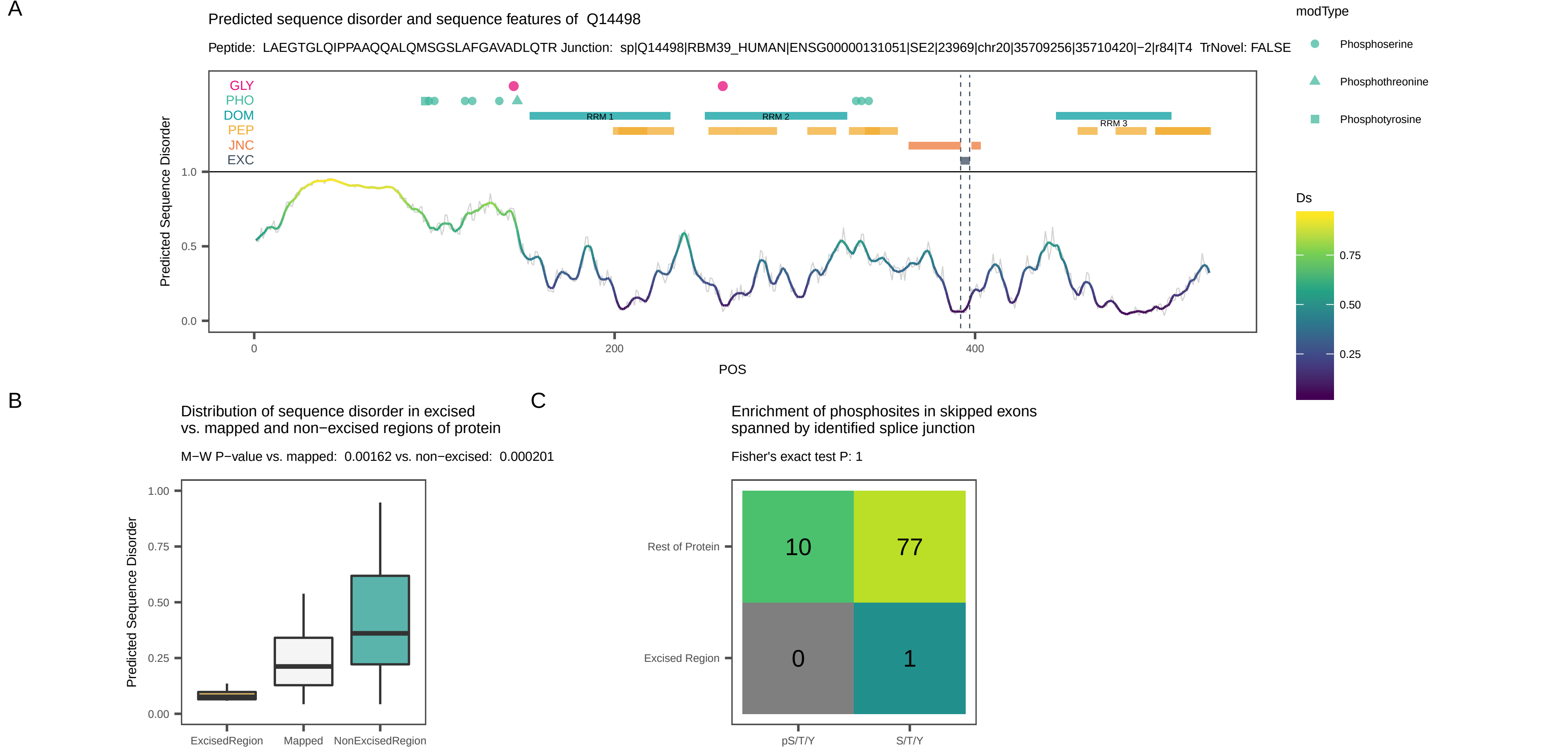
A
Predicted sequence disorder and sequence features of Q14498
Peptide: LAEGTGLQIPPAAQQALQMSGSLAFGAVADLQTR Junction: sp|Q14498|RBM39_HUMAN|ENSG00000131051|SE2|23969|chr20|35709256|35710420|−2|r84|T4 TrNovel: FALSE
GLY
PHO
DOM
PEP
JNC
EXC
RRM 1
RRM 2
RRM 3
Predicted Sequence Disorder
1.0
0.5
0.0
0
200
400
POS
modType
Phosphoserine
Phosphothreonine
Phosphotyrosine
Ds
0.75
0.50
0.25
B
Distribution of sequence disorder in excised
vs. mapped and non−excised regions of protein
M−W P−value vs. mapped: 0.00162 vs. non−excised: 0.000201
Predicted Sequence Disorder
1.00
0.75
0.50
0.25
0.00
ExcisedRegion
Mapped
NonExcisedRegion
C
Enrichment of phosphosites in skipped exons
spanned by identified splice junction
Fisher's exact test P: 1
Rest of Protein
Excised Region
10
77
0
1
pS/T/Y
S/T/Y
